# Development of diagnostic PCR and LAMP markers for *MALE STERILITY 1* (MS1) in *Cryptomeria japonica* D. Don

**DOI:** 10.1186/s13104-020-05296-8

**Published:** 2020-09-29

**Authors:** Yoichi Hasegawa, Saneyoshi Ueno, Fu-Jin Wei, Asako Matsumoto, Tokuko Ujino-Ihara, Kentaro Uchiyama, Yoshinari Moriguchi, Masahiro Kasahara, Takeshi Fujino, Shuji Shigenobu, Katsushi Yamaguchi, Takahiro Bino, Tetsuji Hakamata

**Affiliations:** 1 Forestry and Forest Products Research Institute, Forest Research and Management Organization, Tsukuba, Ibaraki Japan; 2 Graduate School of Science and Technology, Niigata University, Niigata, Japan; 3 Graduate School of Frontier Sciences, The University of Tokyo, Kashiwa, Chiba Japan; 4 National Institute for Basic Biology, Okazaki, Aichi Japan; 5 Shizuoka Prefectural Research Institute of Agriculture and Forestry, Hamamatsu, Shizuoka Japan

**Keywords:** ALP, ASP, LAMP, Molecular markers, Variant detection, *Cryptomeria japonica*, Sugi

## Abstract

**Objective:**

Due to the allergic nature of the pollen of *Cryptomeria japonica*, the most important Japanese forestry conifer, a pollen-free cultivar is preferred. Mutant trees detected in nature have been used to produce a pollen-free cultivar. In order to reduce the time and cost needed for production and breeding, we aimed to develop simple diagnostic molecular markers for mutant alleles of the causative gene *MALE STERILITY 1* (*MS1*) in *C. japonica* to rapidly identify pollen-free mutants.

**Results:**

We developed PCR and LAMP markers to detect mutant alleles and to present experimental options depending on available laboratory equipment. LAMP markers were developed for field stations, where PCR machines are unavailable. The LAMP method only needs heat-blocks or a water bath to perform the isothermal amplification and assay results can be read by the naked eye. Because the causative mutations were deletions, we developed two kinds of PCR markers, amplified length polymorphism (ALP) and allele specific PCR (ASP) markers. These assays can be visualized using capillary or agarose gel electrophoresis.

## Introduction

Molecular markers allow for the selection of specific phenotypes, once the genetic linkage of markers with associated genotypes and candidate gene(s) are identified. Due to the large body mass of tree species, especially conifers, maker-assisted selection with a candidate gene at the early life stage (e.g., seedling) is helpful in reducing the cost and time of breeding. Sugi (*Cryptomeria japonica* D. Don) is the most important forestry conifer in Japan, it occupies about 40% of artificial forests. Due to its fast growth and straight bowl, sugi timber has been used for building materials and for daily life consumables, such as chopsticks and bowls. Recently, however, the pollen of sugi has caused seasonal allergies, which affects approximately 25% of Japanese citizens [1]. Therefore, breeding efforts are currently focused on producing a sterile pollen cultivar [2]. A candidate gene for male sterility, *MALE STERILITY 1* (*MS1*), has been identified by transcriptome analysis and linkage mapping [3–5]. Sequencing analysis of the gene (CJt020762: 2,479 bp) from 83 samples identified 49 haplotypes with observed (*H*_O_) and expected (*H*_E_) heterozygosity of 0.892 and 0.920, respectively (Additional file 1), along with the two deletion mutations, both of which were expected to result in the mutant alleles which cause sterility (*ms1*-*1* and *ms1*-*2* of the haplotype 38 and 4, respectively) [4]. Diagnostic molecular markers are useful for work both in laboratories and forest stations to search for the mutant alleles (*ms1*). Such markers would be useful in breeding efforts to produce pollen-free male sterile sugi.

Here, we developed diagnostic markers for *MS1* in sugi for use in laboratories and forest stations. Amplicon length polymorphism (ALP) and allele specific PCR (ASP) markers were developed for laboratories equipped with capillary and/or gel electrophoretic systems. These markers are suitable for precise determination of genotypes. For work taking place in nurseries and forest stations, we also developed LAMP (loop-mediated isothermal amplification) [6] primer sets. LAMP experiments are less resource intensive and enable the rapid detection of alleles in the field without the need for a molecular biology laboratory. Using these markers in breeding projects for male sterility will boost the production of male sterile cultivars, while simultaneously reducing airborne pollen and mitigating citizens’ allergies.

## Main text

### Materials and methods

Three individual trees with known genotypes for *MS1*: ‘Fukushima1’ (*ms1*-*1*/*ms1*-*1*), ‘Ooi-7’ (*Ms1*/*ms1*-*2*), and ‘G492’ (*Ms1*/*ms1*-*1*) were used from a mapping family of *MS1* [5]. ‘G492’ is an offspring of the cross between ‘Fukushima1’ (male sterile seed parent) and ‘Ooi-7’ (male fertile pollen parent). Sequencing analysis was performed for the *MS1* gene (CJt020762) for ‘Fukushima1’ and ‘Ooi-7’, and it confirmed a 4-base pair (bp) deletion on the first exon and a 30-bp deletion on the third exon, respectively, for the *ms1*-*1* and *ms1*-*2* alleles [4]. An additional sample, ‘Shindai3’ (*ms1*-*1*/*ms1*-*1*), was also analyzed.

We applied the cetyltrimethylammonium bromide (CTAB) method, which is widely used to extract DNA from tree species. CTAB efficiently removes polysaccharides from tree tissue and recovers high-quality DNA [7]. In the current study, about 100 mg of leaves, which were frozen with liquid nitrogen, were ground into fine powder with a TissueLyser II (Qiagen). The powder was washed by adding 0.9 mL of extraction buffer I (Additional file 2) and collected by centrifugation. The supernatant was discarded. This process was repeated if the supernatant was viscous. Then, 0.3 mL of wash buffer (Additional file 2) and 30 μL of 10% sodium N-dodecanoyl salcosinate were added. The mixture was left for 15 min at room temperature, before 0.3 mL of 2 × CTAB buffer (Additional file 2) was added. The mixture was incubated for 10 min. at 60 °C. Then, 0.6 mL of chloroform-isoamylalchol (chloroform:isoamylalchohol = 24:1) was added, the aqueous layer was collected by centrifugation, and the DNA was precipitated by adding 2/3 volume of isopropanol. The precipitate was collected by centrifugation, washed by 70% ethanol, and dissolved into an appropriate volume (100 μL) of TE buffer. RNA molecules were digested by adding 1 μL of RNase (2 mg/mL) and incubating for 2 h at 37 °C. One micro-litter of DNA solution (around 10–50 ng DNA) was used for amplification.

Because the target mutations of *MS1* gene (CJt020762, DNA Databank of Japan, DDBJ, accession numbers: LC536580 and LC538205) are deletions, we designed PCR primers to amplify products of differing length using Primer3 software [8, 9]. PCR was performed in a 10 μL reaction containing 1 μL of template DNA, 1 × Multiplex PCR Master Mix (Qiagen), and 0.2 μM of non-tailed primer, and 0.1 μM of each of tail [10] and tailed primer (Table 1 and Additional file 3). The reaction was initially heat-denatured for 15 min at 95 °C, followed by 35 cycles of 94 °C for 30 s, 60 °C for 90 s, and 72 °C for 60 s, with a final extension for 30 min at 60 °C, using a GeneAmp 9700 PCR System (Applied Biosystems). In addition, we synthesized specific primers to which fluorescent dye was directly attached and then carried out multiplex PCR (Additional file 3) using KAPA2G Fast PCR Kit (NIPPON Genetics). The PCR products were diluted appropriately (usually by 10 times) and run on a 3130 Genetic Analyzer (Applied Biosystems) with LIZ size standard (ThermoScientific).

**Table 1:** Primer sequences for PCR and LAMP diagnostic marker sets of *MS1* in *Cryptomeria japonica*

Maker ID	Primer ID	Primer sequence^a^
(a) ALP_ms1-1	CJt020762_F145	CCATGCCTTTCTTACATGACGAG
	CJt020762_R294_A	GCCTCCCTCGCGCCATTGATTAATGGGAAAGCCCAGAA
(b) ALP_ms1-2	CJt020762_F1O7.F_A	GCCTCCCTCGCGCCACCTCCGGTGTATCAAACTTCAA
	CJt020762_F1O7.R	ATTCGCCCTTTCCAAATGTTAGC
(c) ALP_ms1	CJt020762_ms1-1_F	GCTATAGACTGCACCGACCC
	CJt020762_ms1-1_R	AGCCCAGAAAATCGTCCCTG
	CJt020762_ms1-2_F	GAATCCACCGCCACGACTAT
	CJt020762_ms1-2_R	TGAACTCTGTTTCCATGGCA
(d) ASP_ms1-1	CJt020762_mt-224F_C.f	CTGTGACTCACTGGCCACAGT**A**AC
	Cjt020762common.r	AGAGAGTGATGGTTTGATTAATGGGAAA
(e) ASP_ms1-1_wt	CJt020762_wt-227F_A.f	GTGACTCACTGGCCACAGTCAT**A**AA
	Cjt020762common.r	AGAGAGTGATGGTTTGATTAATGGGAAA
(f) LAMP_ms1-1	4D-1_FIP	ACGATGGGGTCGGTGCAGTCTAGCGGTAGCAGAAAGTGTGAT
	4D-1_BIP-3C	ACGAGATCAGCCAAGCTTTCCAACCCTGCGTGGGT**C**TG
	4D-1_F3	GCGGCAATTGTGAGAGCATT
	4D-1_B3	GAAAATCGTCCCTGGAGACG
(g) LAMP_ms1-1_wt	MS1-190727_FIP-2A	T**A**TTGATGACTGTGGCCAGTGAAGATCAGCCAAGCTTTCCAT
	MS1-190727_BIP	GGTGCTTGTGCGAGCTCGTCGGAGCGAGAGAGTGATGGTT
	MS1-190727_F3	ATCGTGAGCCTCTCACCA
	MS1-190727_B3	GGTCAAGTTCACGCGGATA
(h) LAMP_ms1-2	30D-1-FIP-3T	TG**T**AGTGATCCCCAAAATAGCCGTTAGCATTGCTGGAGCC
	30D-1-BIP	TGAAGTTTGATACACCGGAGGTTTCTGCTAAATATAACTTTGAACTCTG
	30D-1-F3	TGATTCGCCCTTTCCAAAT
	30D-1-B3	AGACAACAGCAATTCATTCA

^a^Bold and underlined fonts indicate artificial mismatch introduced to increase specificity

For laboratories where capillary sequencers are not available, allele specific PCR (ASP) primers, whose products can be analyzed on agarose gels, were designed with Primer3 software [9]. One of the 3’ ends of a primer was positioned on the mismatched site due to the deletion. In addition, an artificial mismatch was introduced at the third base from 3’ end (antepenultimate position) of the primer to increase the difference in the melting temperature between matched and mismatched primer-template pairs. This mismatch increased allelic specificity of the primer [11, 12]. The antepenultimate position is, for the most part, effective to differentiate alleles. Moreover, either purine-purine or pyrimidine-pyrimidine mismatch types, which are expected to be more unstable than that of purine-pyrimidine mismatch, were selected. Multiplex PCR for *MS1* and one of the eight microsatellite markers in *C. japonica* (Additional file 4) were tested, and the best pair was selected based on readability of the amplified bands on agarose gels. Because it is better to include a positive control reaction in PCR, we amplified a microsatellite marker as a positive control. As a result, at least one band was visible when the product was analyzed by electrophoresis. To mitigate non-specific amplification, primers that produce shorter PCR products (with the Primer3 option: “PRIMER_PRODUCT_SIZE_RANGE = 100–250”) and shorter elongation time in PCR were selected. Reactions were carried out in 10 μL reaction containing 1 μL of template DNA, 1 × Multiplex PCR Master Mix (Qiagen), and 0.2 μM of each of microsatellite forward and reverse primer, 0.2 μM of each for both ASP forward and reverse primer (Table 1). The reaction was initially heat-denatured for 15 min at 95 °C, followed by 38 cycles of 94 °C for 15 s, 63 °C for 45 s, and 72 °C for 15 s, using a GeneAmp 9700 PCR System (Applied Biosystems). The PCR products were separated by 2% agarose gel electrophoresis, and stained with ethidium bromide.

Primer sets for LAMP reactions were designed by PrimerExplorer V5 software (https://primerexplorer.jp/e/index.html) with wild and mutant type target sequences based on ‘Fukushima1’ with default parameter setting. Design option “specific” was selected, which allowed us to design allele specific LAMP primers. To increase the specificity of LAMP reactions and discriminate allelic types, an artificial mismatch was introduced at the 3′ or 5′ position of the allele specific primer (the bold and underlined character of the primer sequence in Table 1). LAMP reactions were set up in 25 μL of mixture using a DNA amplification kit (Eiken Chemical Co., Ltd.) according to the manufacture’s instruction. They were incubated at 63 or 65 °C for 120 min, and deactivated at 80 °C for 5 min or 95 °C for 2 min. Fluorescent detection reagent (Eiken) was used to visually check the amplified products. These reactions were carried out by GeneAmp 9700 PCR System (Applied Biosystems) or real-time turbidimeter, LoopampEXIA (Eiken).

## Results

The characteristics of PCR based markers are listed in Table 1. Separation of PCR products on the capillary sequencer clearly showed the length difference (in 4-bp between *ms*-*1* and others and 30-bp between *ms1*-*2* and others). The difference in length enabled genotype identifications (Table 2 and Additional file 3). Moreover, for *Ms1* and *ms1*-*2* alleles, separation on the 2% agarose gels was attained. Products from heterozygous individual (*Ms1*/*ms1*-*2*) showed three bands indicating heteroduplex formation (Additional file 3). A figure describing the ALP and ASP in different genotypes for *MS1* in a blind test trial is provided in Additional file 5.

**Table 2:** Expected and observed results of PCR and LAMP diagnostic marker for *MS1* in *Cryptomeria japonica*

Maker ID	Expected size (bp)^#1^ or presence/absence (±) of products			Observed size (bp) or presence/absence (±) of products		
	*Ms1* (wild allele)	*ms1*-*1* (4-bp deletion)	*ms1*-*2* (30-bp deletion)	*Ms1* (wild allele)	*ms1*-*1* (4-bp deletion)	*ms1*-*2* (30-bp deletion)
ALP_ms1-1	165	161	165	162	158	162
ALP_ms1-2	146	146	116	141	141	112
ALP_ms1	172/288^#2^	168	258	169/286^#2^	165	255
ASP_ms1-1	–	103	–	–	+	–
ASP_ms1-1_wt	105	–	105	+	–	+
LAMP_ms1-1	–	+	–	–	+	–
LAMP_ms1-1_wt	+	–	+	+	–	+
LAMP_ms1-2	–	–	+	+^#3^	+^#3^	+

^#1^Tail sequences of primers are included to calculate the expected PCR product size

^#2^Wild type allele is expected to produce fragments with 172 and 288 bp without 4- and 30-bp deletions, respectively

^#3^Products are still absent after 60 min incubation, but present after 120 min incubation (Additional file 6)

Allele specific PCR amplified the intended products which allowed the differentiation of the presence or absence of the specific bands on agarose gels (Additional file 3). Under the PCR conditions described in the present study, we observed weak bands of positive control (SSR marker: CS1364) for cases where the target allele (wild or mutant allele) was present. PCR reaction mainly amplified the target with shorter PCR products. Similarly, for cases where the target alleles were absent, we observed a strong single band of the positive control, this effectively suppressed the unspecific amplification from the mismatched target.

The LAMP primer sets were first designed with complete template-primer matching for each target allele. When possible, loop primers were also tested to speed up the LAMP reaction. Although inclusion of loop primers shortened the reaction time, they often caused false-positives. Due to these false positives, we used primers with a single artificial mismatch without loop primers. We selected the best allele specific primers (FIP: forward internal primer or BIP: backward internal primer) which showed the fastest amplification (Additional file 6).

## Discussion

We have shown that the mutant alleles for *MALE STERILITY 1* (*MS1*) can be detected in different ways and provided experimental options depending on available laboratory resources. A multiplex PCR reaction can be constructed to simultaneously detect the mutant alleles (*ms1*-*1* and *ms1*-*2*). Moreover, multiplex PCR primer sets, including microsatellite markers, will be useful to manage breeding materials for male sterility. These sets enable the clonal identification of each sample. Using the allele specific primer, we could locate the mutant allele among large genetic resources. Additionally, primers that amplify wild type alleles could be useful in mapping families or breeding materials, where genotyping is necessary.

In field forest stations without PCR machines LAMP reactions are convenient. As far as we know, this is the first report to use LAMP for variant detection in coniferous species with a large genome (10.8 Gbp for *C. japonica* [13]). Our effort mainly focused on the detection of a 4-bp deletion by LAMP because this variant is widespread in our country [5]. In addition, it is hard to separate a 4-bp difference on agarose gel electrophoresis, while a 30-bp difference can be easily separated on 2% agarose gels (Additional file 3). The LAMP methods for *ms1*-*1* are therefore more requested than those for *ms1*-*2*. For LAMP, as for allele specific PCR, the use of artificial mismatch (ARMS-LAMP [14]) increased the specificity, suppressed the amplification from non-target alleles, and increased the speed of amplification for the target allele. However, complete suppression of non-target alleles was difficult (Additional file 3 (h) LAMP_ms1-2). The use of PNA (peptide nucleic acid) [15] to block the alternative allele and to increase the specificity (PNA-LAMP) is an avenue for further study to better refine this method (Additional file 7).

## Limitations

- Multiplex PCR may need optimization, depending on markers (such as microsatellites) combined and PCR machines used.
- LAMP assays may be further optimized by using other primer combinations or applying other methods, such as PNA-LAMP [15]. Sensitivity and specificity of LAMP assay has not been determined, and using both positive and negative controls is necessary to cross-validate the assay. Because we have no homozygous individuals with *ms1*-*2* allele and we did not construct plasmid harboring the allele, the LAMP assay to detect wild type allele without a 30-bp deletion is not reported.
- The reactions are not tested for crude DNA, for which further optimization may be needed.

## Supplementary information

**Additional file 1: Table S1.** Summary of the sequencing analysis for 83 individuals for CJt020762. Number of individual trees (*N*), length of the nucleotide sequence (bp), number of SNPs (nSNP), number of INDELs (nINDEL), number of haplotypes (*nH*), observed (*H*_O_) and expected heterozygosity (*H*_E_). *H*_E_ was calculated by using GenAlEx 6.5 software.

**Additional file 2: Table S2.** Buffer composition used for DNA extraction from *Cryptomeria japonica.* **a** extraction buffer I, **b** wash buffer, and **c** 2 × CTAB buffer.

**Additional file 3: Figure S1.** Reaction manual and representative results for diagnostic markers. The composition of reaction mixture, thermal conditions, and amplification results for each marker. Marker ID is the same as in Table 1 and 2.

**Additional file 4: Table S3.** Microsatellite markers tested in multiplex PCR with the diagnostic ASP markers for *MS1.* Maker ID, fluorescent dye, accession number, SSR motif, expected PCR product size (bp), forward primer sequence, reverse primer sequence, and reference information.

**Additional file 5: Figure S2**. Blind test trial for (**a**) ASP_ms1-1, (**b**) ASP_ms1-1_wt and (**c**) ALP_ms1-2. Twenty-three samples were blind-tested for consistency between the assay and expected genotypes based on sequencing analysis.

**Additional file 6: Figure S3.** Turbidity graph for LAMP assay with *ms1*-*1* specific BIP primers using ‘Shindai3’ (a: *ms1*-*1*/*ms1*-*1*) and ‘Ooi-7’ (b: *Ms1*/*ms1*-*2*) DNA template. The x-axis indicates time in minutes and the y-axis indicates turbidity.

**Additional file 7: Figure S4.** PNA-LAMP assay blind test trial for (**a**) LAMP_ms1-1, (**b**) LAMP_ms1-1_wt. Twenty-three samples were blind-tested for consistency between the assay and expected genotypes based on a sequencing analysis. The detailed protocol is available upon request to the corresponding author.

## Data Availability

The DNA sequences of CJ020762 *ms1*-*1* in ‘Fukushima1’, *Ms1* in ‘Ajigasawa20’ and *ms1*-*2* in ‘Ooi-7’ have been deposited in the DNA Data Bank of Japan (DDBJ) with accession numbers: LC536580, LC538204, and LC538205, respectively (They are available with the following URL: http://getentry.ddbj.nig.ac.jp/getentry/na/xxxxxxxx/, where xxxxxxxx indicates each accession number). The datasets generated during and/or analyzed during the current study are available from the corresponding author on request.

## Abbreviations

ALP: Amplified length polymorphism
ARMS: Amplification refractory mutation system
ASP: Allele specific PCR
BIP: Backward inner primer
bp: Base pair
DDBJ: DNA Data Bank of Japan
DNA: Deoxyribonucleic acid
FIP: Forward inner primer
Gbp: Giga base pair
LAMP: Loop-mediated isothermal amplification
PCR: Polymerase chain reaction
PNA: Peptide nucleic acid
RNA: Ribonucleic acid
