# Supplementary material for: Development of diagnostic PCR and LAMP markers for MALE STERILITY 1 (MS1) in Cryptomeria japonica D. Don
Source: BMC Res Notes. 2020 Sep 29;13:457. doi: 10.1186/s13104-020-05296-8 (PMC7526249; doi:10.1186/s13104-020-05296-8)
Supplement: Supplementary file 3 — Additional file 3: Figure S1. Reaction manual and representative results for diagnostic markers. The composition of reaction mixture, thermal conditions, and amplification results for each marker. Marker ID is the same as in Table 1 and 2. [file 13104_2020_5296_MOESM3_ESM.pdf]

(a) ALP_ms1-1

Amplified fragment length polymorphism (ALP) marker to detect *ms1-1* allele in *MALE STERILITY 1* (*MS1*) in sugi.

Reaction	Volume (μL)
2 × Multiplex (QIAGEN)	3
2 μM CJt020762_F145	1
2 μM CJt020762_R294_A	0.5
2 μM Tail_A	0.5
dH_2O	4
DNA (10—50 ng)	1
Total (μL)	10

Temperature (°C)		Cycles
95	15 min	1
94	15 sec	
63	45 sec	38
72	15 sec	
60	30 min	1
4	∞	1

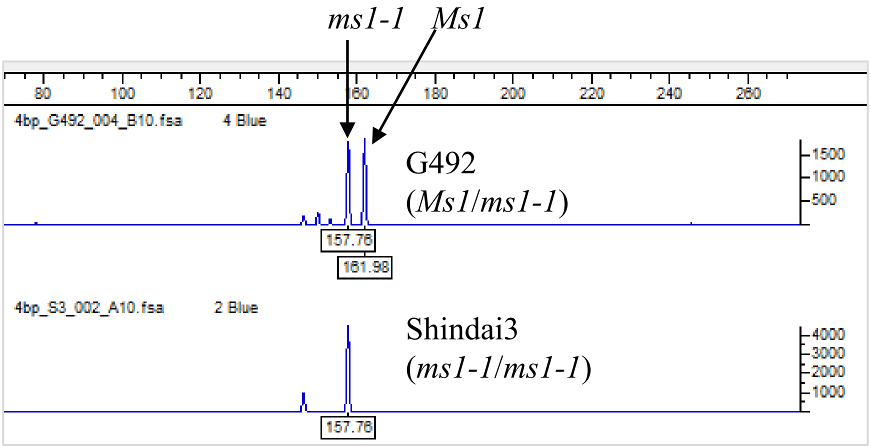

Electropherogram

Primer ID	Primer sequence
CJt020762_F145	CCATGCCTTTCTTACATGACGAG
CJt020762_R294_A	GCCTCCCTCGCGCCATTGATTAATGGGAAAGCCCAGAA
Tail A	[FAM]-GCCTCCCTCGCGCCA

(b) ALP_ms1-2

Amplified length polymorphism (ALP) marker to detect *ms1-2* allele in *MALE STERILITY 1* (*MS1*) in sugi.

Reaction	Volume (μL)
2 × Multiplex (QIAGEN)	3
2 μM CJt020762_F1O7.F_A	1
2 μM CJt020762_F1O7.R	0.5
2 μM Tail_A	0.5
dH_2O	4
DNA (10—50 ng)	1
Total (μL)	10

Temperature (°C)		Cycles
95	15 min	1
94	15 sec	
63	45 sec	38
72	15 sec	
60	30 min	1
4	∞	1

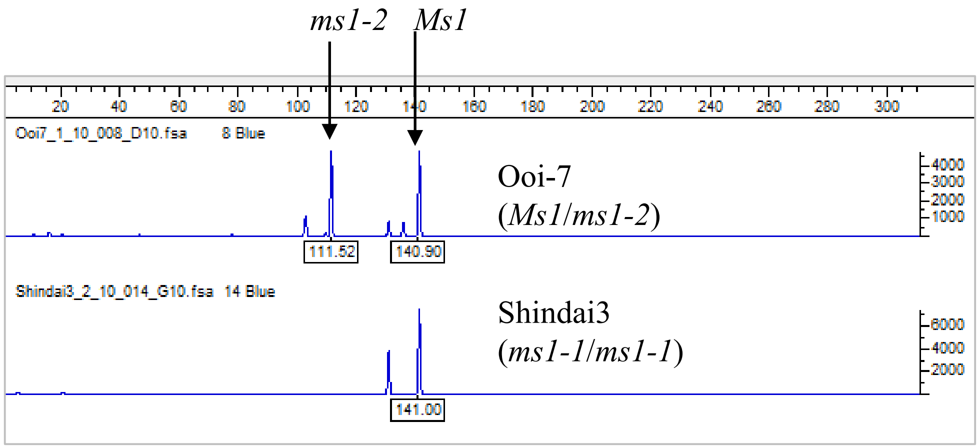

Electropherogram

Primer ID	Primer sequence
CJt020762_F1O7.F_A	GCCTCCCTCGCGCCACCTCCGGTGTATCAAACTTCAA
CJt020762_F1O7.R	ATTCGCCCTTTCCAAATGTTAGC
Tail A	[FAM]-GCCTCCCTCGCGCCA

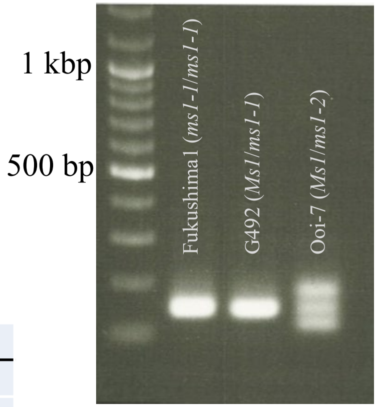

Gel image (2% agarose)

(c) ALP_ms1

Amplified length polymorphism marker (ALP) for *MALE STERILITY 1* (*MS1*) in sugi. Two mutant alleles (*ms1-1* and *ms1-2*) and rest of the wild type alleles (*Ms1*) can be detected simultaneously by multiplex PCR.

Reaction	Volume (mL)
KAPA2G Fast DNA Polymerase (5 U/μL)	0.1
5 × KAPA2G Buffer A[$]	2.0
25 mM $MgCl_2$	0.2
dNTP Mix (10 mM each)	0.2
5 μM CJt020762_ms1-1_F	0.4
5 μM CJt020762_ms1-1_R	0.4
5 μM CJt020762_ms1-2_F	0.2
5 μM CJt020762_ms1-2_R	0.2
dH_2O	5.3
DNA (5 ng/μL)	1.0
Total (mL)	10

[$] 1 × KAPA2G Buffer A contains 1.5 mM $MgCl_2$

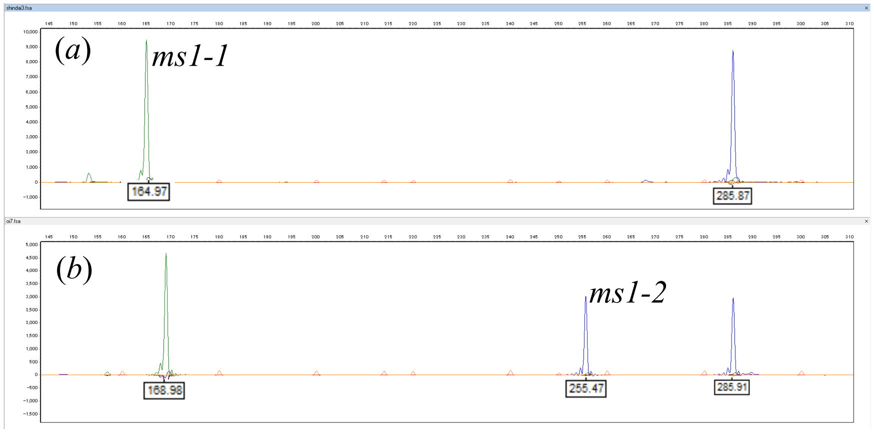

Electropherogram for (a) Shindai3 (*ms1-1*/*ms1-1*) and (b) Ooi-7 (*Ms1*/*ms1-2*)

Temperature (°C)		Cycles
95	3 min	1
95	15 sec	
60	15 sec	35
72	1 sec	
72	1 min	1
4	∞	1

Primer ID	Primer sequence
CJt020762_ms1-1_F	GCTATAGACTGCACCGACCC
CJt020762_ms1-1_R	AGCCCAGAAAATCGTCCCTG
CJt020762_ms1-2_F	GAATCCACCGCCACGACTAT
CJt020762_ms1-2_R	TGAACTCTGTTTCCATGGCA

(d) ASP_ms1-1 and **(e) ASP_ms1-1_wt**

Allele specific PCR reaction to detect *ms1-1* (d) and *Ms1* (e) allele in *MALE STERILITY 1* (*MS1*) in sugi.

Reaction	
2 × Multiplex (QIAGEN)	3
2 µM CJt020762_ASP[$]	1
2 µM Cjt020762common.r	1
2 µM CS1364_F	1
2 µM CS1364_R	1
dH_2O	2
DNA	1
Total (µL)	10

[$] Primer ID for ASP targeting (d) *ms1-1* and (e) *Ms1* is CJt020762_mt-224F_C.f and CJt020762_WT-227F_A.f, respectively.

Temperature (°C)		Cycles
95	15 min	1
94	15 sec	
56	45 sec	38
72	15 sec	
4	∞	1

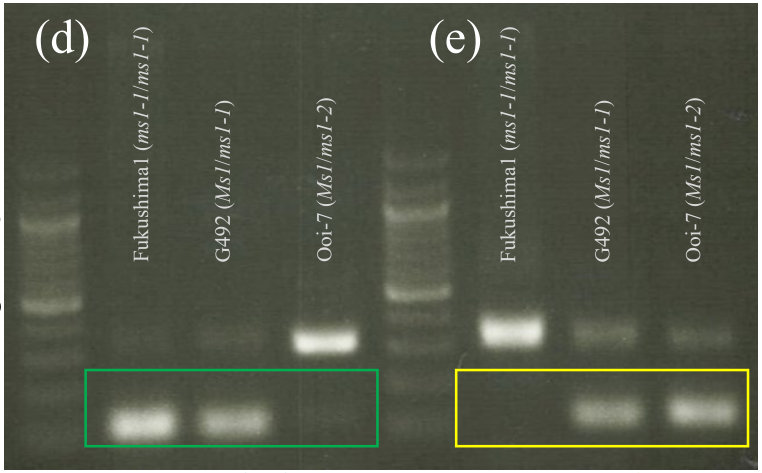

Gel image (2% agarose)

Amplification of CS1364 is suppressed if that of ASP surpasses.

Primer ID	Primer sequence
CJt020762_mt-224F_C.f	CTGTGACTCACTGGCCACAGTAAC
CJt020762_WT-227F_A.f	GTGACTCACTGGCCACAGTCATAAA
CJt020762common.r	AGAGAGTGATGGTTTGATTAATGGGAAA
CS1364_F	TGATTATGGTCGGTGGTCTT
CS1364_R	GTGATGTGGTGTTATCTTGT

(f) LAMP_ms1-1

LAMP reaction to detect *ms1-1* allele in *MALE STERILITY 1* (*MS1*) in sugi.

Reaction	
2 x RM	12.5
40 μM FIP (4D-1_FIP)	1
40 μM BIP (4D-1_BIP-3C)	1
5 μM F3 (4D-1_F3)	1
5 μM B3 (4D-1_B3)	1
Bst DNA polymerase	1
DNA	2
dH_2O	5.5
Total (μL)	25

Temperature (°C)	
63	120 min
80	5 min

Primer ID	Primer sequence
4D-1_FIP	ACGATGGGGTCGGTGCAGTCTAGCGGTAGCAGAAAGTGTGAT
4D-1_BIP-3C	ACGAGATCAGCCAAGCTTTCCAACCCTGCGTGGGTCTG
4D-1_F3	GCGGCAATTGTGAGAGCATT
4D-1_B3	GAAAATCGTCCCTGGAGACG

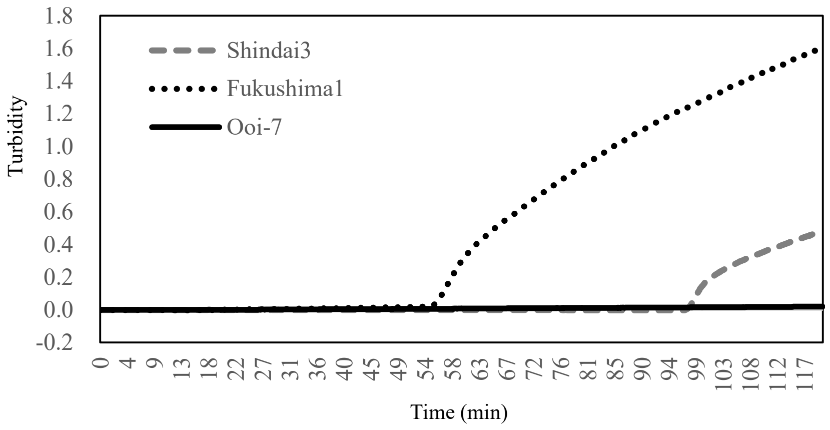

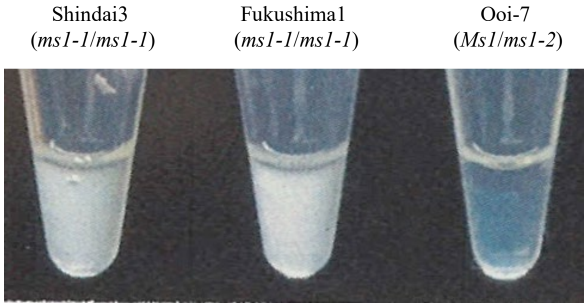

White precipitate was generated after 120 min incubation for assay with *ms1-1*.

(g) LAMP_ms1-1_wt

LAMP marker to detect wild type allele (*Ms1*) without 4-bp deletion.

Reaction	
2xRM	12.5
40 μM FIP (MS1-190727_FIP-2A)	1
40 μM BIP (MS1-190727_BIP)	1
5 μM F3 (MS1-190727_F3)	1
5 μM B3 (MS1-190727_B3)	1
Bst DNA polymerase	1
DNA	2
dH_2O	5.5
Total (μL)	25

Temperature (°C)	
63	120 min
80	5 min

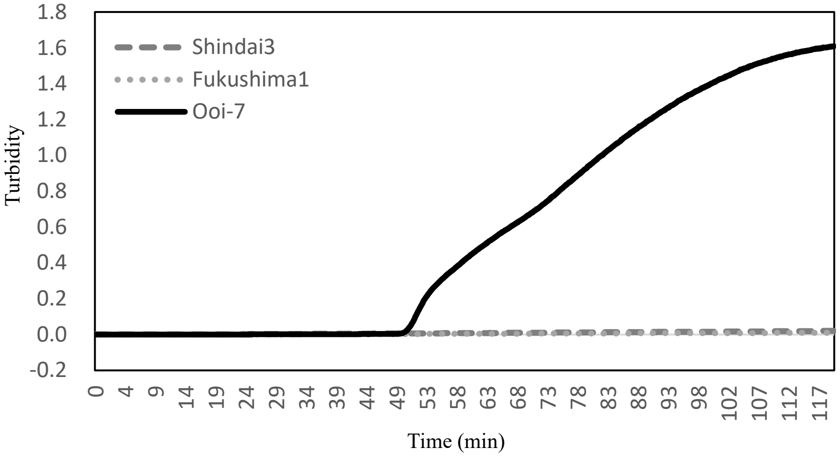

Shindai3 (*ms1-1/ms1-1*) Fukushima1 (*ms1-1/ms1-1*) Ooi-7 (*Ms1/ms1-2*)

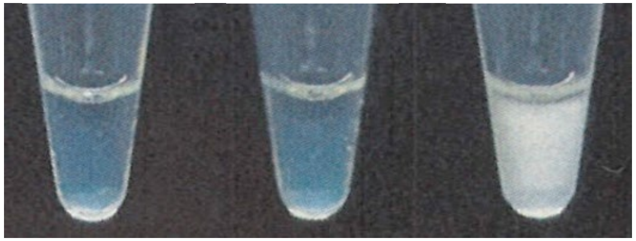

White precipitate was generated after 120 min incubation for assay with *Ms1*

Primer ID	Primer sequence
MS1-190727_FIP-2A	TATTGATGACTGTGGCCAGTGAAGATCAGCCAAGCTTTCCAT
MS1-190727_BIP	GGTGCTTGTGCGAGCTCGTCGGAGCGAGAGAGTGATGGTT
MS1-190727_F3	ATCGTGAGCCTCTCACCA
MS1-190727_B3	GGTCAAGTTCACGCGGATA

(h) LAMP_ms1-2

LAMP reaction to detect *ms1-2* allele in *MALE STERILITY 1* (*MS1*) in sugi.

Reaction	
2xRM	12.5
40 μM FIP (30D-1-FIP-3T)	1
40 μM BIP (30D-1-BIP)	1
5 μM F3 (30D-1_F3)	1
5 μM B3 (30D-1_B3)	1
Bst DNA polymerase	1
DNA	2
dH_2O	5.5
Total (μL)	25

Temperature (°C)	
65	120 min
80	5 min

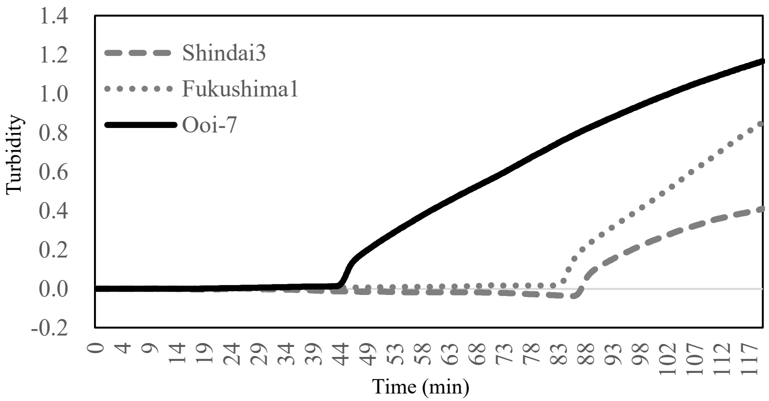

Shindai3 (*ms1-1*/*ms1-1*) Fukushima1 (*ms1-1*/*ms1-1*) Ooi-7 (*Ms1*/*ms1-2*)

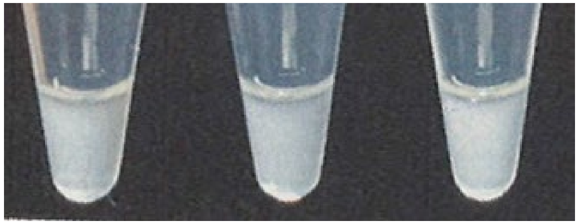

White precipitate was generated after 120 min incubation for all assays. Inclusion of positive control and stopping the reaction at 60 min. is necessary.

Primer ID	Primer sequence
30D-1-FIP-3T	TGTAGTGATCCCCAAAATAGCCGTTAGCATTGCTGGAGCC
30D-1-BIP	TGAAGTTTGATACACCGGAGGTTTCTGCTAAATATAACTTTGAACTCTG
30D-1-F3	TGATTCGCCCTTTCCAAAT
30D-1-B3	AGACAACAGCAATTCATTCA
