# Supplementary material for: Development of diagnostic PCR and LAMP markers for MALE STERILITY 1 (MS1) in Cryptomeria japonica D. Don
Source: BMC Res Notes. 2020 Sep 29;13:457. doi: 10.1186/s13104-020-05296-8 (PMC7526249; doi:10.1186/s13104-020-05296-8)
Supplement: Supplementary file 5 — Additional file 5: Figure S2. Blind test trial for (a) ASP_ms1-1, (b) ASP_ms1-1_wt and (c) ALP_ms1-2. Twenty-three samples were blind-tested for consistency between the assay and expected genotypes based on sequencing analysis. [file 13104_2020_5296_MOESM5_ESM.pdf]

Figure S2 Blind test trial for (A) ASP_ms1-1, (B) ASP_ms1-1_wt and (C) ALP_ms1-2 Twenty-three samples were blind-tested for consistency between the assay and expected genotype based on sequencing analysis.

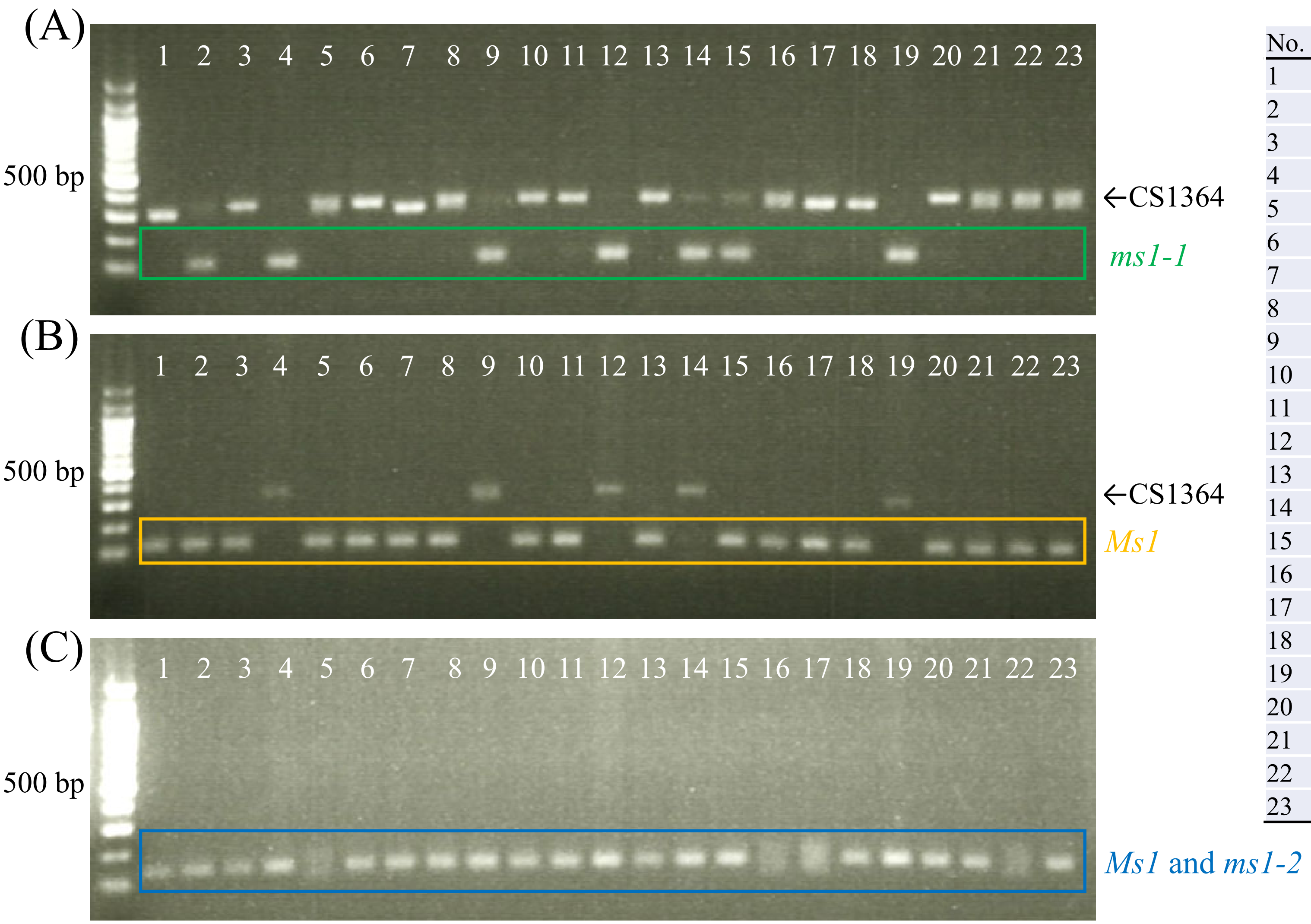

No.	Tree ID	genotype
1	Ashu14	*Ms1/Ms1*
2	Suzu-2	*Ms1/ms1-1*
3	Ashu13	*Ms1/Ms1*
4	Shindai-12	*ms1-1/ms1-1*
5	Ishinomaki07	*Ms1/ms1-2*
6	Ajigasawa31	*Ms1/Ms1*
7	Ajigasawa33	*Ms1/Ms1*
8	Azouji25	*Ms1/Ms1*
9	Fukushima-1	*ms1-1/ms1-1*
10	Azouji34	*Ms1/Ms1*
11	Ashu17	*Ms1/Ms1*
12	Shindai-11	*ms1-1/ms1-1*
13	Ashu01	*Ms1/Ms1*
14	Shindai-3	*ms1-1/ms1-1*
15	Naka-4	*Ms1/ms1-1*
16	Ishinomaki10	*Ms1/ms1-2*
17	Ooi-7	*Ms1/ms1-2*
18	Ajigasawa20	*Ms1/Ms1*
19	Fukushima-2	*ms1-1/ms1-1*
20	Bijodaira14	*Ms1/Ms1*
21	Azouji22	*Ms1/Ms1*
22	Ishinomaki04	*Ms1/ms1-2*
23	Azouji24	*Ms1/Ms1*
