# Supplementary material for: Development of diagnostic PCR and LAMP markers for MALE STERILITY 1 (MS1) in Cryptomeria japonica D. Don
Source: BMC Res Notes. 2020 Sep 29;13:457. doi: 10.1186/s13104-020-05296-8 (PMC7526249; doi:10.1186/s13104-020-05296-8)
Supplement: Supplementary file 6 — Additional file 6: Figure S3. Turbidity graph for LAMP assay with ms1-1 specific BIP primers using ‘Shindai3’ (a: ms1-1/ms1-1) and ‘Ooi-7’ (b: Ms1/ms1-2) DNA template. The x-axis indicates time in minutes and the y-axis indicates turbidity. [file 13104_2020_5296_MOESM6_ESM.pdf]

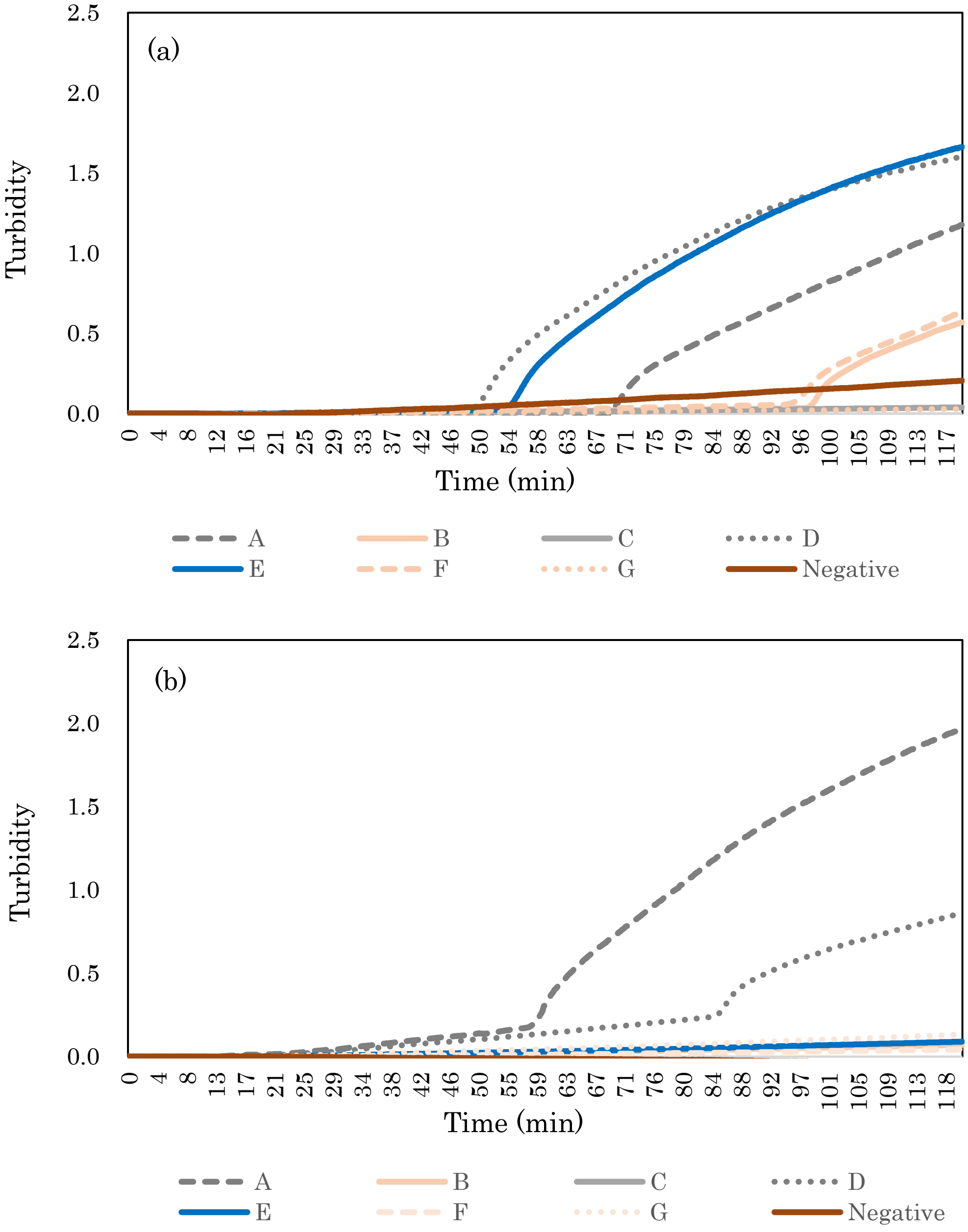

Figure S3 Turbidity graph for LAMP assay with *ms1-1* specific BIP primers using 'Shindai3' (a: *ms1-1*/*ms1-1*) and 'Ooi-7' (b: *Ms1*/*ms1-2*) DNA template.

Each BIP primer has its own mismatched base at 3' position, with CON-XXX. CON indicates the constant part (ACGAGATCAGCCAAGCTTTCCAACCCTGCGTGGGTCTG) and XXX indicates variable bases. XXX for A to G, respectively, are GTG, GAG, GGG, GCG, CTG, ATG, TTG, where the underline indicates mismatched base introduced artificially. The BIP primer E (4D-1_BIP-3C in Table 1), shown by the blue line, shows the preferential amplification for *ms1-1*/*ms1-1* and no amplification for the *Ms1*/*ms1-2* sample. This indicates the excellent specificity of primer E for *ms1-1* compared to other primers.
