# Supplementary material for: Development of diagnostic PCR and LAMP markers for MALE STERILITY 1 (MS1) in Cryptomeria japonica D. Don
Source: BMC Res Notes. 2020 Sep 29;13:457. doi: 10.1186/s13104-020-05296-8 (PMC7526249; doi:10.1186/s13104-020-05296-8)
Supplement: Supplementary file 7 — Additional file 7: Figure S4. PNA-LAMP assay blind test trial for (a) LAMP_ms1-1, (b) LAMP_ms1-1_wt. Twenty-three samples were blind-tested for consistency between the assay and expected genotypes based on a sequencing analysis. The detailed protocol is available upon request to the corresponding author. [file 13104_2020_5296_MOESM7_ESM.pdf]

Figure S4 Blind test trial for (A) PNA-LAMP_ms1-1_wt, and (B) PNA-LAMP_ms1-1
Twenty-three samples were blind-tested for consistency between the assay and expected genotypes based on a sequencing analysis. The detailed protocol is available upon request to the corresponding author.

(A)

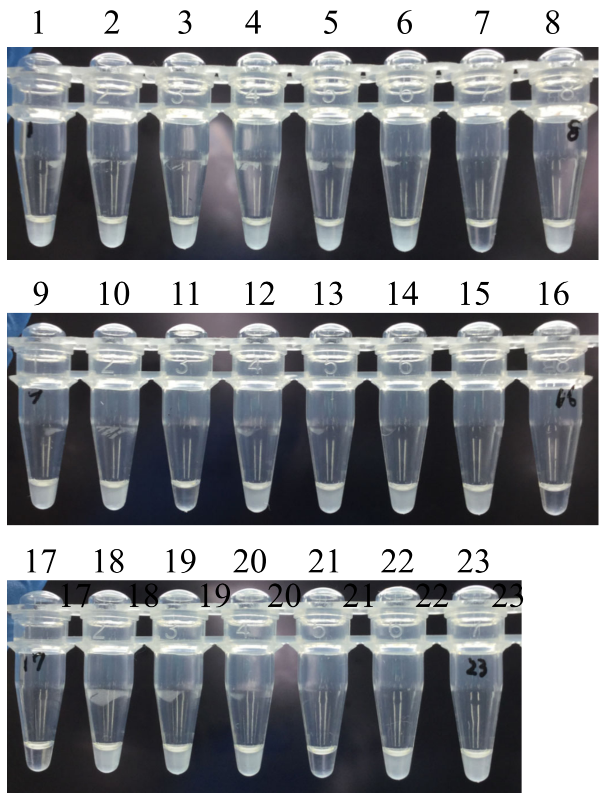

(B)

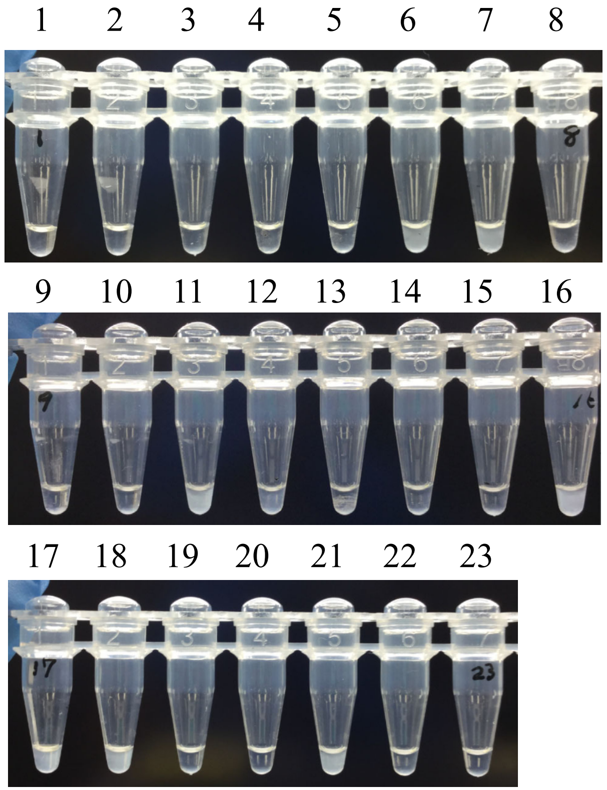

No.	Tree ID	genotype
1	Bijodaira14	*Ms1/Ms1*
2	Ashu13	*Ms1/Ms1*
3	Azouji34	*Ms1/Ms1*
4	Ishinomaki04	*Ms1/ms1-2*
5	Ashu14	*Ms1/Ms1*
6	Naka-4	*Ms1/ms1-1*
7	Shindai-11	*ms1-1/ms1-1*
8	Ajigasawa33	*Ms1/Ms1*
9	Ashu17	*Ms1/Ms1*
10	Azouji22	*Ms1/Ms1*
11	Shindai-3	*ms1-1/ms1-1*
12	Azouji25	*Ms1/Ms1*
13	Ashu01	*Ms1/Ms1*
14	Ishinomaki10	*Ms1/ms1-2*
15	Ajigasawa31	*Ms1/Ms1*
16	Fukushima-1	*ms1-1/ms1-1*
17	Shindai-12	*ms1-1/ms1-1*
18	Suzu-2	*Ms1/ms1-1*
19	Azouji24	*Ms1/Ms1*
20	Ooi-7	*Ms1/ms1-2*
21	Fukushima-2	*ms1-1/ms1-1*
22	Ishinomaki07	*Ms1/ms1-2*
23	Ajigasawa20	*Ms1/Ms1*
